# Multifaceted Tissue-Protective Functions of Polyvalent Immunoglobulin Preparations in Severe Infections—Interactions with Neutrophils, Complement, and Coagulation Pathways

**DOI:** 10.3390/biomedicines11113022

**Published:** 2023-11-10

**Authors:** Carolin Schmidt, Sabrina Weißmüller, Corina C. Heinz

**Affiliations:** 1 Department of Corporate Clinical Research and Development, Biotest AG, 63303 Dreieich, Germany; 2 Department of Preclinical Research, Biotest AG, 63303 Dreieich, Germany

**Keywords:** bacterial infection, coagulation, human polyvalent immunoglobulins, neutrophils, sCAP, sepsis, tissue damage

## Abstract

Severe infections induce immune defense mechanisms and initial tissue damage, which produce an inflammatory neutrophil response. Upon dysregulation of these responses, inflammation, further tissue damage, and systemic spread of the pathogen may occur. Subsequent vascular inflammation and activation of coagulation processes may cause microvascular obstruction at sites distal to the primary site of infection. Low immunoglobulin (Ig) M and IgG levels have been detected in patients with severe infections like sCAP and sepsis, associated with increased severity and mortality. Based on Ig’s modes of action, supplementation with polyvalent intravenous Ig preparations (standard IVIg or IgM/IgA-enriched Ig preparations) has long been discussed as a treatment option for severe infections. A prerequisite seems to be the timely administration of Ig preparations before excessive tissue damage has occurred and coagulopathy has developed. This review focuses on nonclinical and clinical studies that evaluated tissue-protective activities resulting from interactions of Igs with neutrophils, complement, and the coagulation system. The data indicate that coagulopathy, organ failure, and even death of patients can possibly be prevented by the timely combined interactions of (natural) IgM, IgA, and IgG with neutrophils and complement.

## 1. Introduction

For severe infections such as severe community-acquired pneumonia (sCAP) and sepsis, depending on the pathogen, guidelines recommend treatment with antibiotics or antiviral agents [1,2,3]. However, adjunctive treatments that support the host’s immune system may still be required. Polyvalent immunoglobulins (Igs) are a long-discussed and promising therapy option for severe infections. Examples of polyvalent Igs include intravenous immunoglobulin G (IVIg) preparations containing ≥95% polyvalent immunoglobulin (Ig)G such as Flebogamma DIF (Grifols, Barcelona, Spain), KIOVIG (Takeda, Vienna, Austria), Intratect (Biotest Dreieich, Germany), Gammagard (Baxter, Illinois, USA), Gamimune N (Bayer, Leverkusen, Germany), or Privigen (CSL Behring, Marburg, Germany), and polyvalent Ig preparations such as Pentaglobin and trimodulin (Biotest, Dreieich, Germany) containing high concentrations of IgM and IgA in addition to IgG. 

The use of Igs as a potential therapeutic option is based, on the one hand, on the biological properties and modes of action of polyvalent Igs and, on the other hand, on the studies that have shown beneficial effects of Igs in different subsets of patients. However, a statistically significant effect of polyvalent Ig treatment on mortality rates has not been demonstrated in most of these studies. This may be due to the limitations some of these studies had, such as a small sample size, not being powered to meet the primary endpoint, a heterogeneous target population, and the use of different types of Ig preparations and/or dose levels.

This review explores the various modes of action of polyvalent Igs. Nonclinical and clinical studies have evaluated the different host-supporting activities of Igs in tissue protection, particularly in the lung and the vasculature, and are outlined here. An overview is also provided on how Igs protect tissues by interacting with neutrophils, complement factors, and the coagulation system, which in turn leads to faster pathogen control and helps in reducing inflammation. Implementing the considerations that are discussed in this review may help in designing future clinical trials with the aim of better supporting the hypothesis that treatment with polyvalent Ig preparations is valuable as an adjunctive treatment option, which not only results in clinical benefit but also in statistically significant effects on the essential clinical parameters in severe infections.

## 2. Endogenous Immunoglobulin Levels in Severe Infections

An important rationale for treatment with Igs is to supplement patients’ endogenous Ig concentrations. Although the relationship between hypogammaglobulinemia and survival has not always been clearly demonstrated [4,5,6,7], several studies have reported a relationship between low Ig plasma levels and an increased risk of mortality in patients with severe infections [8,9,10,11]. The decrease in Ig plasma levels may be attributed to an increased Ig consumption due to infection (e.g., increased neutrophil-dependent phagocytosis and natural killer cell-dependent antibody-dependent cellular cytotoxicity), vascular leakage caused by endothelial cell dysfunction, excessive catabolism, impaired Ig production by B-cells, and increased sepsis-related apoptosis of lymphocytes causing lymphopenia [12,13]. Indeed, sepsis non-survivors appeared to have reduced circulating numbers of antibody-producing B lymphocytes 24 h after sepsis onset compared with non-septic patients and sepsis survivors [14]. Depletion of B lymphocytes (e.g., detectable as lymphopenia) could contribute to the immunosuppression mechanisms induced by severe bacterial infections [12].

Although there is limited clinical evidence that the decrease in Ig plasma levels is transient, normalization of Ig plasma levels over the course of the disease has been shown [4,10,15], and a progressive increase in Ig plasma levels over time was seen in patients who survived [16]. Other studies showed that Ig levels (particularly IgM and IgG) decrease with disease severity in patients with community-acquired pneumonia, sCAP, sepsis, and septic shock [17,18,19,20]. Although direct proof is still pending, the available data from patients with immunodeficiency suggest that there is a higher risk of infection when Ig levels are constitutively low before the infection. In addition, otherwise healthy individuals with normal Ig levels at the time of infection can develop low plasma Ig levels during the course of the disease, which then contributes to the severity of the disease. 

Several clinical studies have demonstrated that IgM levels varied with disease severity at the time of diagnosis of severe sepsis, septic shock, community-acquired pneumonia, or sCAP (bacterial or viral), and low levels of IgM seem to be associated with an increased risk of mortality [11,18,20,21,22,23,24]. For example, low IgM serum concentrations have been associated with uncontrolled proinflammatory responses and mortality in sCAP that was caused by a pandemic influenza virus [21].

With respect to IgG, an observational study in patients with sCAP showed that low levels of total IgG, IgG1, and IgG2 were significantly associated with higher 30-day mortality (*p* < 0.001, *p* = 0.004, *p* = 0.016, respectively) [17,25]. This relationship was confirmed in other studies in patients with septic shock [8,9]. A relationship between low total IgG and/or IgG2 levels and disease severity was also observed in other studies in patients with sepsis, septic shock, or severe pneumonia (bacterial or viral) [15,20,24,26,27]. In one study, low IgG levels were associated with significantly higher 28-day mortality in patients with sepsis or septic shock compared with patients who had normal IgG levels (*p* < 0.001) [26].

So far, only a few studies have been published that show a decrease in IgA plasma concentrations in severely infected patients. IgA primarily acts in the mucosa and, therefore, a decrease is not easy to assess in plasma. In the case of respiratory failure, it is possible that tissue damage may result in the leakage of IgA from mucus or alveoli into the blood stream, which may increase plasma IgA concentrations [4]. Although transient IgA deficiency in plasma was observed in a low percentage of patients suffering from sCAP, systemic inflammatory response syndrome, or septic shock, IgA serum levels were also inversely associated with the probability of death [8,9].

### Polyvalent Immunoglobulins and Pathogen-Induced Tissue Damage

**Figure 1:**
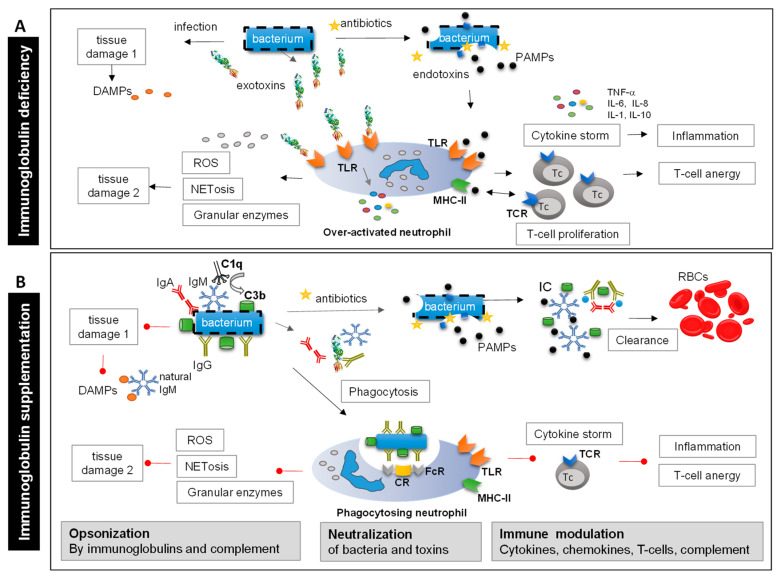
Immune responses in bacterial infections during Ig deficiency and Ig supplementation. (**A**) In the absence of sufficient amounts of Igs, tissue damage (release of DAMPs) can be caused by either bacteria or through secretion of bacterial exotoxins and release of endotoxins (i.e., PAMPs), for instance, after antibiotic treatment. DAMPs and PAMPs both lead to an inflammatory response followed by recruitment of neutrophils. Due to stimulation with toxins, PAMPS, and cytokines, neutrophils secrete ROS and proteases. Overstimulation might result in NETosis, causing secondary tissue damage. The cytokine storm and damaged tissues further fuel persistent inflammation. Persistent pro- and anti-inflammatory responses and overstimulation (proliferation) of T-cells may lead to pathological T-cell responses (e.g., apoptotic depletion, decreased proliferation, and T helper 2 cell polarization and unresponsiveness (T-cell anergy)). (**B**) In the presence of sufficient amounts of Igs, bacteria and their toxins as well as dead bacteria and disintegrated bacterial particles (i.e., PAMPs) and damaged tissue cells and particles (i.e., DAMPs) are opsonized with antibodies. In the presence of immune IgM, natural IgM, and IgG, additional complement opsonization facilitates fast clearance of bacteria, their PAMPs, and DAMPs via local phagocytosis by neutrophils and/or via transport on the RBCs to phagocytes in the liver. This prevents overstimulation of neutrophils, persistent inflammation, tissue damage, and immune suppression. C1q: complement factor C1q; C3b: complement factor C3b; CR: complement receptor; DAMPs: damage-associated molecular patterns; FcR: Fc receptor; IC: immune complexes; Ig(s): immunoglobulin(s); IL: interleukin; MHC-II: major histocompatibility complex II; NET: neutrophil extracellular trap; PAMPs: pathogen-associated molecular patterns; RBCs: red blood cells; ROS: reactive oxygen species; Tc: cytotoxic T-cell; TCR: T-cell receptor; TLR: toll-like receptor; TNF-a: tumor necrosis factor alpha.

Low Ig levels may lead to decreased pathogen clearance, which may increase pathogen-induced tissue damage. Figure 1A summarizes the different pathways that may lead to tissue damage, and Figure 1B summarizes how Igs can help mitigate tissue damage. Igs protect tissues directly by efficiently binding to bacteria, thereby blocking the tissue-damaging activity of endotoxins and exotoxins (Figure 1A). Binding of Igs to bacteria (including multi-drug-resistant bacterial strains) and toxins supports neutralization and clearance of these immune complexes via phagocytosis (Figure 1B). The IgM present in an IgM/IgA-enriched Ig preparation enhances additional complement deposition on the pathogen, thereby facilitating more efficient opsonophagocytosis (i.e., uptake of pathogens bound by antibodies and complement) and clearance. If polyvalent Igs are given early during the course of infection, the extent of infection- or toxin-induced local tissue damage may be restricted, which may prevent the development of excessive inflammation [28]. Details on the characteristics and functions of IgM, IgG, and IgA, including interactions with pathogens during severe infections, and on the different polyvalent Ig preparations (IVIg and IgM/IgA-enriched preparations) have been summarized elsewhere [28].

**In summary**, low levels of polyvalent IgM and IgG in plasma and/or IgA in mucosa may affect the course of the disease and the survival of patients with severe infections. This implies that treatment with Ig preparations containing IgM, IgA, and/or IgG may have the potential to favorably influence disease outcome, particularly in patients with low Ig levels, by raising Ig levels back to normal reference ranges. This might, therefore, be a relevant rationale for Ig therapy. 

## 3. Polyvalent Immunoglobulins and Inflammation-Induced Tissue Damage

### 3.1. The Role of Immunoglobulins in the Modulation of Cytokine-Induced Inflammation

During severe infections, inflammation is characterized by excessive cytokine release, which is primarily triggered by activation of Toll-like receptors (TLRs, e.g., TLR1, TLR2, TLR4) on the neutrophil cell surface. TLRs are activated through the binding of pathogens, toxins, and bacterial debris (i.e., pathogen-associated molecular patterns (PAMPs)). Opsonization of pathogens with Igs, fast pathogen clearance, and exotoxin neutralization prevent PAMPs binding to TLRs and reduce TLR signaling, leading to attenuated cytokine/chemokine secretion. Several retrospective and prospective clinical studies demonstrated a reduction of cytokine levels and other acute-phase protein levels (C-reactive protein (CRP), procalcitonin (PCT)) in the plasma of septic patients after treatment with standard IVIg [29,30,31,32,33,34] or IgM/IgA-enriched preparations [35,36,37,38,39,40].

In addition to the above, cytokine release can be reduced through decreased expression of the TLR. Several studies have shown this effect following administration of IgM/IgA-enriched Ig or standard IVIg preparations [41,42,43]. When both preparations were compared in vitro in a neutrophil/severe acute respiratory syndrome coronavirus-2 (SARS-CoV-2) model system of coronavirus disease 2019 (COVID-19)-associated inflammation, both preparations reduced inflammation [44,45]. However, the IgM/IgA-enriched Ig preparation elicited stronger Fcγ receptor (FcγR)- and Fcα receptor 1-dependent immune modulation and cytokine secretion by neutrophils. In addition, it has been shown that Igs can bind directly to cytokines, thus inhibiting cytokine activity (“scavenging”), and to Ig receptors on the neutrophils, thus modulating cytokine secretion [46,47,48,49].

### 3.2. The Role of Immunoglobulins in the Modulation of Complement-Induced Inflammation

The complement system is required to induce opsonophagocytosis of complement factor (C)3b- and C4b-opsonized pathogens by phagocytes and to induce cytotoxic effects, resulting in cell lysis of the pathogen and anaphylatoxin release. This pathway is dependent on the binding of Igs to the pathogen. In severely infected patients with low Ig levels, modulation of complement activity is likely impaired, which may fuel inflammation.

Polyvalent Igs have a dual, concentration-dependent function in the complement pathway. IgM is the main Ig isotype that modulates complement activity in a concentration-dependent manner. It is the most effective Ig isotype to activate the complement cascade. It enhances complement deposition, thereby enhancing opsonophagocytosis by neutrophils and macrophages. At higher concentrations, however, IgM interacts with already-formed complement fragments (“scavenging”), thereby attenuating their activity [50,51,52,53,54,55,56,57]. Based on weight and molar basis, monomeric IgM is superior to IgG and IgA at inhibiting the uptake of activated complement fragments in antigen–antibody immune complexes on cell surfaces [51,58]. IVIg at high concentrations can also divert complement activation products from target surfaces, but without blocking activation per se [59]. These interactions with the complement pathway occur further downstream and attenuate anaphylatoxin activity and excessive bacterial cell lysis (i.e., complement-dependent cytotoxicity (CDC)) via the terminal complement cascade [50,51,55,57]. Previous studies using high doses of IVIg have shown that complement scavenging dampens the proinflammatory effects of anaphylatoxins [57,60].

Pathogen-specific IgM is the primary host-immune response to infection. High plasma levels of IgM are found during the early stages of infection. The endogenous switch from bacterial clearance via opsonophagocytosis at high IgM concentrations to clearance via CDC at low IgM concentrations suggests that it might be a problem if severely infected patients develop low Ig levels [55]. This may lead to high anaphylatoxin generation due to increased clearance of pathogens via CDC and thus an increase in complement-induced inflammation. 

Limiting anaphylatoxin activity during infection seems important to limit inflammation [61]. Anaphylatoxins cause the impairment of neutrophil phagocytosis activity and the respiratory burst of neutrophils, which may lead to neutrophil degranulation [62,63,64] and the further release of their contents as neutrophil extracellular traps (NETs, see Section 4.1.1) [65,66]. These neutrophils may die subsequently (NETosis). Furthermore, high levels of C5a not only induce vasodilatation and contribute to vascular permeability, but also cause homing of neutrophils to the microvasculature instead of the tissues. The described processes can lead to vascular inflammation associated with NETs, obstruction and reduction of microcirculatory blood flow, ischemia, hypoxia, tissue damage, and organ failure [67].

The importance of controlling anaphylatoxin activity was shown in studies with patients who died from sepsis. In these patients, C3a and C4a plasma levels were considerably elevated, which correlated with disease severity [68]. Similarly, in a prospective study in patients with sepsis, the presence of high C5a levels was associated with reduced expression levels of C5a receptors (C5aR and C5L2) and reduced production of interleukin (IL)-8 in neutrophils, which was associated with a poor prognosis [69]. In other prospective studies, high levels of anaphylatoxins were associated with mortality [68,70]; it was discussed that complement may play a role in multiple organ failure (MOF). Surviving septic patients had lower anaphylatoxin levels than non-survivors [71,72]. In addition to bacterial infections, anaphylatoxins were associated with inflammation and acute lung injury in severe viral infections, including influenza, severe acute respiratory syndrome, and COVID-19 [73,74,75,76,77]. Limiting anaphylatoxin activity in infection with the aim of reducing inflammation is important. For this reason, several C5a receptor inhibitors are currently in clinical development for the treatment of COVID-19, vasculitis, and other autoimmune and inflammatory diseases [61,78,79,80].

### 3.3. The Role of Natural IgM in Modulation of DAMP-Induced Inflammation 

During infection, inflammation can also be triggered by the excessive release of biomolecules that are released by damaged cells of the host (i.e., damage-associated molecular patterns (DAMPs), danger signals, or alarmins). DAMPs include components of damaged, infected, or dying host tissue cells and are believed to activate pattern recognition receptors of innate immune cells (e.g., TLR2, TLR4, and TLR6 on neutrophils), leading to inflammatory responses. Interestingly, natural IgM is important to avoid this process [81,82]. Natural IgM is generated in the absence of stimulation by specific antigens, whereas immune IgM is generated following exposure to pathogens [83,84,85]. Natural IgM is produced by B1-cells and marginal zone B lymphocytes and has low affinities but broad specificities to both foreign and self-structures [86,87]. It recognizes oxidation-specific epitopes such as oxidized phosphatidylcholines present in the membranes of apoptotic cells. The binding of natural IgM to apoptotic cells and, consequently, their clearance via (opsono)phagocytosis is required to prevent the release of autoantigens and proinflammatory factors and the progression to secondary necrosis [88,89,90].

The functions of natural IgM were investigated using the natural IgM antibody LR04, which binds to oxidation-specific epitopes present on microvesicles [91]. Microvesicles are a class of cell membrane-derived extracellular particles that are released upon cellular stress in inflammatory processes [92]. Vesicles containing negatively charged phospholipids may bind to coagulation factor X/Xa, thus facilitating prothrombinase complex formation, and consequently have been found to contribute to thrombosis [93,94]. Natural IgM prevented microvesicle-induced coagulation in vitro, and intravenous injection of natural IgM protected mice from microvesicle-induced pulmonary thrombosis [95]. The large pentameric IgM molecules prevented the binding of factor X/Xa to the microvesicle surface by steric hindrance. This mode of action may be relevant for bacterial sepsis or severe viral infections such as COVID-19, where patients are at higher risk of developing thrombosis.

In patients with stroke, post-stroke lung and urinary tract infections may develop, which are associated with increased morbidity and mortality [96]. Using an experimental mouse model, it was shown that stroke is related to a depletion of marginal B2 cells and a reduction of circulating natural IgM concentrations [97]. Treatment of mice with an IgM/IgA-enriched Ig preparation enhanced bacterial clearance from the lung after induction of stroke [98]. Furthermore, normalization of splenic plasma B-cell numbers and endogenous mouse IgM and IgA concentrations and a reduction in proinflammatory cytokines in the lung were observed. 

IgM/IgA-enriched polyvalent Ig preparations are expected to contain predominantly natural IgM because it is present in the source plasma pools from healthy donors [81]. As discussed here, the natural IgM component in these preparations is expected to play an additive role in the prevention of excessive activation of TLRs on neutrophils by binding to DAMPs [99,100]. Preparations containing polyvalent natural and immune IgM may therefore be more effective or more diverse in their ability to reduce DAMP-induced inflammatory responses than IVIg preparations.

**In summary**, treatment with standard IVIg or IgM/IgA-enriched preparations results in the modulation of inflammatory responses by various mechanisms. Not only are endogenous cytokine and acute-phase protein levels reduced, but also inflammation caused by pathogens, their toxins, and other PAMPs. Modulation is provided by IgG and is complemented by IgA and IgM. IgG blocks the Fc receptor and modulates cell receptors, whereas specific IgG present in polyvalent preparations provides anti-cytokine activity and anaphylatoxin scavenging. IgA provides for additional anti-pathogenic activity and immunomodulation on mucosal surfaces, including those in the respiratory tract. Immune IgM modulates complement activity, thereby supporting opsonophagocytosis and attenuating anaphylatoxin activity. Natural IgM limits DAMP-induced inflammation processes. Severely infected patients who have low IgM levels may thus particularly benefit from treatment with IgM/IgA-enriched Ig preparations.

## 4. Indirect Tissue-Protective Functions of Polyvalent Immunoglobulins

### 4.1. Alveolar Damage in Severe Lung Infection

**Figure 2:**
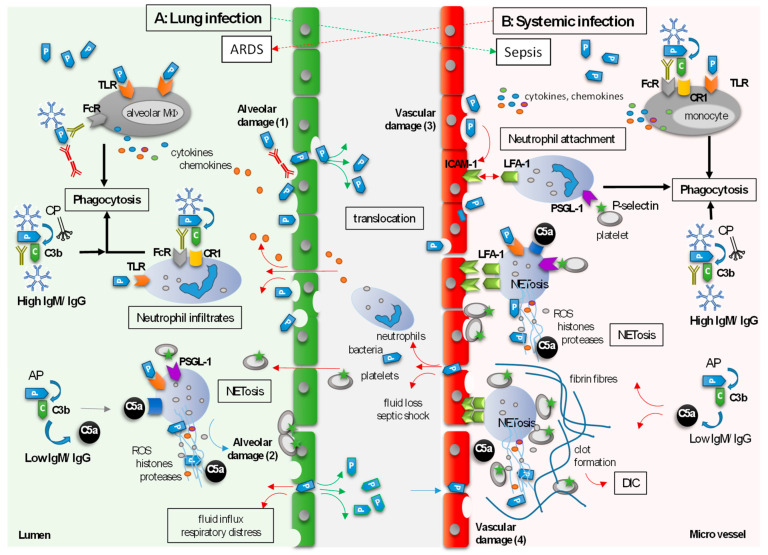
Neutrophil and Ig actions at the epithelial/endothelial barrier of alveoli leading from lung infection to sepsis or from systemic infection to ARDS. (**A**) In the case of lung infection, bacteria (P) enter the lung alveoli (green area), where they are phagocytosed by alveolar macrophages. Due to infection-related damage of alveolar epithelium (1, green lining) and activation of macrophages, cytokines and chemokines (colored circles) are secreted. Neutrophils and platelets translocate from the vasculature (red lining) through the interstitium (gray area) into the alveoli. Neutrophils support phagocytosis, which is enhanced in the presence of polyvalent Igs (high IgM/IgG) and when bacteria are complement-opsonized (via CP). In the absence of Igs, particularly IgM (low IgM/IgG), complement is activated by the AP causing release of C5a (anaphylatoxin). Overactivation of neutrophils by bacteria (TLR stimulation), platelets, and C5a, leads to NETosis, which further damages the alveoli (2, green lining). Tissue damage facilitates translocation of bacteria into the blood stream (green arrows) causing sepsis. (**B**) When bacteria disseminate into the vasculature, they are phagocytosed by monocytes. Phagocytosis is enhanced in the presence of IgG, IgA, and IgM, and after additional complement deposition. If, however, bacterial load is too high, they cannot be sufficiently cleared and may damage endothelial cells (3, red lining). Platelets are activated and plug the damaged vessel wall. In addition, activated endothelial cells express adhesion molecules such as ICAM-1. Secretion of cytokines and chemokines attract and activate neutrophils that attach to the endothelium, e.g., via integrin LFA-1 to ligand ICAM-1. Activated platelets express P-selectin and engage with the P-selectin receptor (via the ligand PSGL-1) on neutrophils. In the absence of sufficient Ig levels (low IgM/IgG), neutrophils are overactivated by binding of PSGL-1 and C5a to their respective receptors and via TLR stimulation inducing NETosis of the attached neutrophils. C5a is generated via the AP and can induce NETs. This leads to activation of coagulation processes and ultimately to immunothrombosis, DIC, and to further vascular damage (4), thus facilitating translocation of bacteria into the interstitium and lung alveoli, causing ARDS (red arrows at the red and green lining). AP: alternative pathway; ARDS: acute respiratory distress syndrome; C: complement; C3b: complement factor C3b (anaphylatoxin); C5a: complement factor C5a (anaphylatoxin); CP: classical pathway; CR1: complement receptor 1; DIC: disseminated intravascular coagulation; FcR: Fc receptor; ICAM-1: intercellular adhesion molecule 1; Ig: immunoglobulin; LFA-1: lymphocyte function-associated antigen 1; MΦ: macrophage; NET: neutrophil extracellular trap; P: bacteria; PSGL-1: P-selectin glycoprotein ligand 1; ROS: reactive oxygen species; TLR: Toll-like receptor.

Despite a decrease in mortality rates over the last few decades, diseases such as sCAP, acute lung injury (ALI), or acute respiratory distress syndrome (ARDS) are still associated with significant morbidity and mortality [101,102]. Pathogens, overactivated neutrophils, and invasive mechanical ventilation may cause damage to the alveoli. While neutrophils are recruited via the blood stream and invade the alveoli, pathogens and cytokines may translocate from the alveoli via the extravascular compartment (i.e., interstitium) into the blood circulation, causing systemic infection, vascular inflammation, and coagulation processes that can harm the host (Figure 2A). Vice versa, systemic infections originating from a distant primary site can be a predisposing factor for pulmonary inflammation and the development of ALI/ARDS (Figure 2B).

#### 4.1.1. The Role of Neutrophils and NETs 

Neutrophil influx into the lung is a defining characteristic not only of the host defense mechanism against pathogens but also of the alveolar damage that occurs in sCAP and ALI/ARDS [103]. During lung infection, circulating neutrophils are attracted by chemokines that are secreted by TLR-activated alveolar macrophages and infected alveolar cells, and by anaphylatoxins that are released through complement activation (Figure 2A). 

In the case of persistent severe infection and insufficient phagocytosis of pathogens (e.g., due to Ig deficiency and/or complement consumption), excessive amounts of bacterial toxins and debris may cause further release of pro- and anti-inflammatory cytokines and chemokines by neutrophils and cause primary lung tissue injury [103]. Furthermore, due to interactions of cytokines, anaphylatoxins, and bacterial toxins with multiple receptors present on neutrophils, and also possibly due to the lack of antibodies required for efficient phagocytosis, neutrophils release NETs [104,105]. NETs are built from chromatin and antibacterial proteins, which can interact with complement and coagulation factors [104]. They contain antimicrobial proteins and enzymes such as myeloperoxidase, elastase, and cathepsin G [106]. With the chromatin fibers, NETs capture pathogens, rendering them innocuous and preventing the spread of pathogens [107]. NETs also contain histones, which extracellularly appear to be a new class of highly tissue-damaging nuclear proteins [108,109,110,111,112,113,114,115,116].

Extensive tissue trafficking, secretion of antimicrobial factors such as reactive oxygen species, antimicrobial peptides, and multiple proteinases—which also help to degrade the extracellular matrix during migration—as well as NETosis of neutrophils can cause secondary tissue injury [117,118]. Hallmarks of secondary lung tissue injury include an increase in lung epithelial and vascular permeability, which leads to an influx of protein-rich edema fluid in the alveoli and arterial hypoxemia [119].

#### 4.1.2. The Role of Immunoglobulins

As discussed in Section 3 and in Weißmüller et al. [28], Igs could have protective functions that may prevent primary alveolar damage (Figure 2B). Polyvalent Igs neutralize exotoxins, PAMPs, and DAMPs via opsonization and effectively induce the Fc receptor/complement receptor-dependent (opsono)phagocytosis. This in turn reduces further cytokine and chemokine secretion, exotoxin-induced tissue damage, and consequently, platelet activation (see Section 5.1). In addition, IgM can dose dependently interact with complement components C3b and C4b to attenuate the terminal complement pathway and reduce anaphylatoxin activity, whereas IgG can directly neutralize C3a and C5a via F(ab)_2_ interaction [57]. By reducing chemokine secretion and the activity of anaphylatoxins, Igs may prevent the recruitment and tissue trafficking of large numbers of neutrophils into the alveoli and might reduce the relevant signals for NETs and NETosis. Consequently, Igs may also prevent secondary alveolar damage caused by neutrophils (Figure 2A, Section 4.1.1). 

A novel mechanism by which IgM may prevent tissue damage was described in a study in the murine cecal ligation puncture (CLP) model. The apoptosis inhibitor of macrophage (AIM) effector protein (also called cluster of differentiation 5-like molecule) was found to be involved in tissue injury, amplified inflammation, increased bacteremia, and increased mortality [120]. Blocking the AIM protein significantly increased the survival of animals with experimental sepsis, decreased local and systemic inflammation, reduced tissue injury, and inhibited bacterial dissemination in the blood, particularly at later time points. Electron microscopy revealed that pentameric IgM binds to the AIM protein [121,122]. This mechanism is of interest for future research. 

Thus, the different modes of action exerted by sufficient amounts of polyvalent Igs not only seem to reduce pathogen- and toxin-induced tissue damage, but may also prevent damage induced by overactivation of neutrophils and, possibly, by blocking AIM activity.

#### 4.1.3. Nonclinical Studies Investigating the Supporting Role of Polyvalent Immunoglobulin in Alveolar Protection

The role of polyvalent Igs in protecting alveolar tissues during infection was investigated in a study where rabbits were first infused with lipopolysaccharide (LPS), followed by Ig infusion starting 15 min after LPS infusion and by *Escherichia coli* intravenous infection 30 min after LPS infusion [123]. Histological samples of rabbit lung specimens were analyzed for pathological features of diffuse alveolar damage. Treatment with an IgM/IgA-enriched Ig preparation significantly improved six of seven diffuse alveolar damage score parameters within 3 h of *Escherichia coli* infection, including alveolar edema, interstitial edema, hemorrhage, inflammatory infiltration, epithelial destruction, and micro-atelectasis. 

In a rat CLP sepsis model, the efficacy of an IgM/IgA-enriched Ig preparation was compared with erythropoietin with respect to alveolar and intestine tissue damage [124]. Erythropoietin is known to suppress the release of tumor necrosis factor (TNF)-α and IL-6, decrease organ damage, and improve the survival rates of rats after hemorrhagic shock [125]. In the control group, Ates et al. observed a disruption of alveolar structure, thickening of the alveolar septa, and inflammatory cell infiltration in the lungs, including intense inflammation of the lamina propria, mucosal shedding, cell infiltration, vascular congestion, hemorrhage, and destruction of the small intestine villi [124]. In rats treated with an IgM/IgA-enriched Ig preparation or erythropoietin before sepsis induction, the histopathologic appearance of the lung and small intestine tissues was normal. The protective effects were more pronounced in rats treated with an IgM/IgA-enriched Ig preparation than in erythropoietin-treated animals.

The influence of Igs on alveolar damage was also investigated in a rat ALI/ARDS model [126]. Primary damage to the alveoli-capillary membrane was induced by shear forces, characterized by loss of barrier functions and rupture of the alveolar epithelium and endothelium, ultimately enabling the translocation of pathogens. Prophylactic treatment of rats with an IgM/IgA-enriched Ig preparation reduced lung lavage-induced translocation of *Klebsiella pneumonia* from the lung into the blood stream; this effect was dose dependent. 

In another study, mice were infected intratracheally with a lethal dose of *Pseudomonas aeruginosa* and treated with increasing doses of IVIg either before infection or 3 h after infection [127]. Compared with the albumin control group (100% lethality), a dose-dependent increase in survival rates was observed in the IVIg groups (0% lethality in the first 24 h in the highest dose group of 2.5 mg IVIg). Lung edema, bacterial load in the lungs, bacteremia, and TNF-α decreased significantly in IVIg-treated mice compared with the control group (*p* < 0.05). Lung histology showed significantly reduced infiltration of inflammatory cells and alveolar hemorrhage compared with the control group. 

In a murine CLP sepsis model, the treatment effect of IVIg on mortality and the development of ALI were investigated [128]. Mice were dosed with 100 and 400 mg/kg IVIg and both doses significantly improved survival (30% and 50%, respectively) compared with the control group (0%). IVIg also reduced plasma levels of IL-6 and TNF-α. ALI (lung injury scores for four parameters—congestion, edema, inflammation, and hemorrhage) was significantly lower than in the control group. Apoptosis of alveolar epithelial cells decreased only with 400 mg/kg IVIg, suggesting a dose-dependent effect.

The effects of IVIg treatment on viral lung infections were investigated in ferrets exposed to either the pandemic H1N1/09 virus or the highly pathogenic avian influenza H5N1 virus [129]. A marked reduction in the number of virally infected lung sites was observed when IVIg was provided as an intravenous prophylaxis; this reduction was not observed when it was provided intranasally. Survival rates increased dose dependently. Most animals treated with IVIg survived a viral dose that was lethal to control animals. It should be noted that the IVIg preparation was derived from plasma of donors who had not been previously exposed to the avian influenza H5N1 virus. The Fab antibody fragment, most likely from a specific cross-reactive antibody, appeared to be responsible for this antibody-mediated survival benefit. 

The in vivo studies in animals described above suggest that administration of IgM/IgA-enriched Ig and IVIg preparations might either prevent or at least mitigate infections, and the formation or deterioration of alveolar injuries at the epithelial and endothelial barrier of the lung that are caused by pathogens and toxins.

#### 4.1.4. Clinical Studies Investigating the Supporting Role of Polyvalent Immunoglobulins in Alveolar Protection 

In order to translate measurements of lung protective properties of polyvalent Igs from nonclinical experiments to the clinic, the number of ventilator days or ventilator-free days, recovery time, mortality rates, and/or hospitalization/intensive care unit (ICU) time can be used as surrogate endpoints. However, it should be noted that these parameters vary with the disease studied (e.g., sCAP, sepsis, septic shock, postoperative sepsis), disease severity (i.e., disease stage) at the time of treatment start, ICU protocols, comorbidities, and other factors. When treatment, for instance, was started too late and patients had already received mechanical ventilation for an extended period of time, Igs did not seem to provide an additional benefit. Therefore, the efficacy results based on surrogate endpoints obtained from several small clinical studies conducted with IVIg and IgM/IgA-enriched Ig preparations have shown heterogeneous results. Most studies were not powered to show a statistically significant difference between the treatment groups for these endpoints. Nevertheless, available clinical studies indicate that the protection of alveoli seems to be an important mechanism of action. 

##### Studies with IVIg in sCAP

IVIg (and hyperimmune IVIg) have been used to treat viral lung infections. One clinical study in patients with sCAP caused by influenza A (H1N1) virus showed that hyperimmune IVIg treatment significantly reduced viral load (Day 5: 3.3 vs. 4.67 log 10 copies/mL; *p* = 0.04, Day 7: undetectable vs. 4.53 log 10 copies/mL; *p* = 0.02) in the lungs compared with IVIg. This protective effect was related to a significantly reduced mortality rate in the hyperimmune IVIg group compared with the IVIg group (overall: 23.5% vs. 29.4% and in a subgroup receiving early treatment: 0% vs. 40%; odds ratio (OR) = 0.14; 95% confidence interval (CI) 0.02–0.92; *p* = 0.04) [130]. Results from this study are consistent with nonclinical data [129].

In contrast, in an observational, matched-cohort study in sCAP patients with septic shock, no difference was noted in ventilator-free days or mortality between the IVIg and the control groups (Table 1, [131]). The authors attributed these observations to (1) the time point of treatment might have been too late (i.e., patients had sCAP that was too advanced) and (2) the treatment dose might have been too low (5 g/day for 3 days). These points are relevant to consider for future studies. 

A dose dependency was also observed in meta-analyses in adult patients with severe sepsis, septic shock, or COVID-19 [132,133,134,135]. An overall reduction in mortality after adjunctive treatment with IVIg appeared to be more pronounced when higher doses (total dose of 1.5–2 g/kg in sepsis and 0.4–1 g/kg/day in COVID-19) were used. This is supported by a study in hospitalized patients with severe COVID-19 who were treated with 20 g/day of IVIg (Table 1, [136]). In this study, the percentage of patients requiring a ventilator was significantly reduced when treatment was given within 48 h of ICU admission compared with patients who were treated >48 h of admission (6.7% vs. 32.1%, *p* = 0.016). This was related to a significantly shorter hospital/ICU stay and a reduction in mortality (Table 1, [136]). Although clinical benefit was also observed in other studies with IVIg preparations (high dose: 20–25 g/day) in patients with COVID-19 [137,138], no clear survival benefit was observed in the other COVID-19 studies [139]. In addition to dosage, the small sample size and advanced disease stage may have contributed to the observed lack of IVIg-related effects on alveolar tissue protection, resulting in an overall survival benefit.

##### Studies with IgM/IgA-Enriched Ig Preparations in sCAP

IgM/IgA-enriched Ig preparations have been investigated in a few studies in patients with sCAP. In a retrospective study in 12 patients with sCAP and steroid-resistant severe acute respiratory syndrome [140], chest radiographic scores and oxygen requirement improved significantly (*p* < 0.05) after treatment with the IgM/IgA-enriched Ig preparation (Table 1, [140]). During the early phase of the COVID-19 pandemic, one case report described clinical amelioration with improved lung performance and lung computerized tomography (CT) scan, increased arterial partial pressure of oxygen/inspired oxygen concentration ratio, and a progressive decrease in inflammatory markers CRP, IL-6, and fibrinogen after treatment with an IgM/IgA-enriched Ig preparation [141]. A case series of 15 patients with severe acute respiratory syndrome coronavirus infection (*n* = 5 on invasive mechanical ventilation) treated with an IgM/IgA-enriched Ig preparation indicated a beneficial effect in patients with lung CT scores < 18, as was shown by markedly improved lung CT scans 4 weeks after admission (Table 1, [142]). Patients with lung CT score > 18 did not seem to have benefited from treatment. A larger randomized, controlled clinical trial in patients with severe COVID-19 treated with IgM/IgA-enriched Ig preparations showed a trend towards a reduced composite deterioration/mortality rate (*p* = 0.096) when treatment was started before invasive mechanical ventilation and before respiratory dysfunction and coagulopathy had progressed into advanced stages [143]. A similar benefit with IgM/IgA-enriched Ig preparations was observed in other studies in COVID-19 patients with less advanced disease (Table 1, [144]) [144,145].

One prospective, randomized clinical trial was performed in a relatively homogenous population of patients with sCAP to evaluate the efficacy of an IgM/IgA-enriched Ig preparation (Table 1, [23]). Treatment started early after the start of invasive mechanical ventilation. A post hoc analysis performed in a subgroup of patients with clear signs of inflammatory disease (CRP ≥ 70 mg/L) showed that the mean number of ventilator-free days was markedly higher in the IgM/IgA-enriched Ig preparation subgroup (mean: 12.1 days; median: 14.0 days; 95% CI 9.8–14.4 days) than the placebo group (mean: 9.2 days; median: 4.0 days; 95% CI 6.8–11.6 days) (*p* = 0.044). Similar results were obtained in patients with IgM baseline levels ≤ 0.8 g/L (*p* = 0.031) or both high CRP/low IgM (*p* = 0.031). The mortality rate was also markedly significantly reduced in the CRP ≥ 70 mg/L subgroup (*p* = 0.030) and in the IgM ≤ 0.8 g/L subgroup (*p* = 0.043) and the combined subgroup (*p* = 0.006), which was clinically meaningful [23].

##### Studies with IVIg Preparations in Sepsis

A small, prospective, non-interventional study in 21 patients with septic shock suggested that patients who have low IgG levels develop ALI/ARDS more frequently (10/12 patients) than patients who have normal IgG levels (3/9 patients) [8]. Although clinical data with standard IVIg preparations in sepsis are sparse, the outcome of the non-interventional study seems to point to a potential protective role for IgG in the prevention of progression to alveolar damage in patients with sepsis. 

One of the largest placebo-controlled IVIg trials published is the Score-Based Immunoglobulin Therapy of Sepsis study, conducted with a broad spectrum of patients, with most patients having medically and surgically severe sepsis and septic shock (Table 1, [146]). Patients received a low dose of IVIg (0.6 g/kg on Day 0; 0.3 g/kg on Day 1). Neither the 7-day mortality rate nor the 4-day pulmonary function improved significantly at the evaluated time points. Nevertheless, the authors concluded that the results obtained with the low dose do not exclude the possibility that efficacy may be observed with a higher dose of IVIg in sepsis [146]. Similar to previous studies that failed to show a benefit of IVIg, the results obtained in this study may be explained by the low IVIg dose used for treatment and/or the inclusion of patients with too advanced disease who are unlikely to benefit from IVIg treatment. It should be noted that a total dose of 1.5–2 g/kg seems to be optimal for administration in septic patients, and the difference in doses was one of the key covariates explaining the majority of the heterogeneity in treatment effects observed across the studies [132,133].

##### Studies with IgM/IgA-Enriched Ig Preparations in Sepsis

In one small, before–after cohort clinical trial (*n* = 26), a statistically non-significant but notable reduction in the number of patients requiring invasive mechanical ventilation after treatment with an IgM/IgA-enriched Ig preparation was observed on Day 4 (IgM/IgA-enriched Ig preparation: 6/12 patients, control: 11/13 patients) [147].

In a randomized, prospective study, late-stage patients who developed septic shock with MOF after pneumonia, sepsis, pancreatitis, cardiac arrest, or ARDS were investigated [38]. The IgM/IgA-enriched Ig preparation showed a statistically not significant but notable reduction in the number of ventilator days (median: 13 days (interquartile range (IQR): 8–20 days) vs. control: 17 days (IQR: 6–29 days)) and the length of stay in the ICU (median: 15 days (IQR: 9–30 days) vs. control: 26 days (IQR: 9–34 days)). This study included a small and highly heterogeneous population with different underlying diseases. In addition, most (70–75%) of these severely ill patients had died during the study (IgM/IgA-enriched Ig preparation: 12/16 patients vs. control: 12/17 patients), which may indicate that patients had a disease stage where it was too late for treatment, again pointing to an early treatment window for Ig therapies. 

In a randomized, prospective study in patients with postoperative sepsis, treatment with an IgM/IgA-enriched Ig preparation shortened the total time of invasive mechanical ventilation for IgM/IgA-enriched Ig preparation-treated patients (mean ventilator days: 9.9 days (range: 1–22 days)) compared with the control group (12.8 days (range: 6–30 days)) (Table 1, [148]). This was related to a marked reduction in the length of hospitalization, and the mortality rate was also markedly reduced (IgM/IgA-enriched Ig preparation: 44.4% vs. control: 76.5%). 

In another randomized controlled trial in ICU patients with postoperative severe infections from pneumonia, septicemia, peritonitis, and wound sepsis, the mean number of ventilator days in the high-risk surgery group (mean: 5.5 days) was significantly (*p* ≤ 0.01) lower for patients who received an IgM/IgA-enriched Ig preparation than the control group (mean: 12.7 days) (Table 1, [149]). Patients with pneumonia in the control group remained in intensive care longer than patients with pneumonia receiving the IgM/IgA-enriched Ig preparation (*p* ≤ 0.01), which showed that recovery was positively influenced by the IgM/IgA-enriched Ig preparation. 

In a retrospective study in patients with septic shock, treatment with an IgM/IgA-enriched Ig preparation markedly increased the number of ventilator-free days (median: 22 days (IQR: 5–28 days) vs. control: 12 days (IQR: 0–26 days)), which was related to an increase in ICU-free days (Table 1, [150]); although clinically relevant, this increase was not statistically significant. In contrast, 30-day mortality was statistically significantly lower in the IgM/IgA-enriched Ig preparation group than in the control group (25.0% vs. 46.1%, *p* = 0.004). 

In a randomized, double-blind study in patients with abdominal infections, patients treated with an IgM/IgA-enriched Ig preparation had a shorter duration of mechanical ventilation, although these data were not reported. This was related to a shorter hospital stay than in patients treated with the albumin control (Table 1, [35]).

**Table 1:** Effect of Igs on alveoli and lung function.

REF	Indication	Ig Preparation (Dose)	N	Effect	Significant Difference	Marked Change	No Difference	Study Results
[140]	SARS-CoV	IgM/IgA-enriched Ig preparation (5 mL/kg/day for 3 consecutive days)	12	Lung scores	X			Treatment with Ig improved recovery from SARS-CoV infection, despite deterioration after initial steroid and ribavirin therapy. Scores derived from chest radiographs were based on the percentage of area involved, manifested as ground glass opacification, consolidation, or nodular shadow in each lung. Compared with Day 1, lung scores significantly improved on Days 5, 6, and 7 (*p* < 0.05) and oxygen requirement significantly improved on Days 6 and 7 (*p* < 0.05) after Ig treatment.
[142]	Severe and critical COVID-19	IgM/IgA-enriched Ig preparation (5 mL/kg/day for 3 consecutive days)	15	Lung CT scores		X		Treatment with Ig improved recovery in SARS-CoV-2-infected patients based on lung CT data. CT scoring was based on lung involvement of each lobe, including ground glass opacity, crazy paving, and consolidation. Improvement in CT scores was observed in nine of ten patients who received Ig and were not on IMV. Treatment was not beneficial in patients already receiving IMV at the start of Ig treatment, 4 of 5 died. Patients with a lung CT score of ≥18 had an almost 4-fold increased risk of death versus those with lower scores.
[144]	Severe and critical COVID-19	IgM/IgA-enriched Ig preparation (variable dosages)	316	VFD			X	Overall, no difference in the number of VFD (*n* = 182 received IMV at baseline) on Day 30 was observed with Igs and mortality rates were similar compared with control (28.8% vs. 31.8%). This was most likely caused by the heterogeneity of the treatment regimen and study population, including severe and critical COVID-19 patients. An improved, nearly statistically significant, benefit in 30-day survival (*p* = 0.063) was observed in a subgroup of patients receiving a high-dose Igs (>15 g/day for >3 days) and who were not on IMV at baseline (*p* = 0.068).
				SURsg		X		
[23]	sCAP	IgM/IgA-enriched Ig preparation (186.2 mg/kg/day for 5 days)	160	VFD			X	Overall, there was no statistically significant difference in VFDs between Ig treatment and control (mean: 11.0 vs. 9.6 days). Post hoc subset analyses in patients with CRP ≥ 70 mg/L (*n* = 124), IgM ≤ 0.8 g/L (*n* = 111), and high CRP/low IgM (*n* = 92) at baseline, showed a marked increase in the number of VFD (e.g., mean 12.6 vs. 8.7 days in the combined high CRP/low IgM subgroup, *p* = 0.031) and significant reductions in all-cause mortality compared with control (e.g., 11.8% vs. 36.6% in the combined subgroup, *p* = 0.006).
				VFDsg	X			
				MORsg	X			
[131]	sCAP receiving IMV and with septic shock	IVIg (5 g/day for 3 days) (dose regimen in Japan)	1324	VFD			X	Overall, there was a non-significant difference in the number of VFD in the Ig group compared with the control (8.3 vs. 9.4 days). No improvement was observed in the propensity sm (*n* = 1045) analysis (8.7 vs. 9.1 days). Similarly, no differences were found in 28-day mortality rates (37.8% vs. 35.3% overall and 36.7% vs. 36.0% in the sm groups). For the dose used in Japan clinically, no benefit was observed in pneumonia patients on IMV with septic shock.
				VFDsm			X	
				MORsm			X	
[136]	Severe COVID-19	IVIg (20 g/day, total dose if ≤48 h: 64.4 ± 54.7 g, and if >48 h: 88.6 ± 71.1 g)	58	IMVsg	X			All patients were treated with Igs which were provided ≤48 h or >48 h after admission. In the subgroup receiving Igs early, the proportion of patients requiring IMV was significantly lower than in the >48 h group (6.7% vs. 32.1%). This positive effect was related to a statistically significant difference in 28-day mortality between the two groups (23.3% vs. 57.1%), a shorter length of hospital stay (11.5 ± 1.0 vs. 17.0 ± 1.6 days), and an ICU stay (9.5 ± 1.1 vs. 13.5 ± 1.6 days).
				MORsg	X			
				HOSsg	X			
[38]	Septic shock with severe respiratory failure	IgM/IgA-enriched Ig preparation (5 mL/kg/day over 3 days)	33	INF	X			Although CRP levels were significantly reduced in the Ig group on Days 4, 5, and 6, no significant difference in the median length of IMV and length of ICU stay was detected. Nevertheless, a trend was observed for the number of VD (median: 13 days vs. control: 17 days) and the length of stay in the ICU (median: 15 days vs. 26 days). See also Table 3.
				VD		X		
				ICUd		X		
[148]	Sepsis after surgery	IgM/IgA-enriched Ig preparation (5 mL/kg daily over 3 days)	35	IMV		X		Although a higher proportion of patients received IMV in the Ig group at baseline, the proportion of patients requiring IMV during the study increased slightly (83.3% to 88.8%) in the Ig group but markedly in the control group (64.7% to 100%). Additionally, the number of VD and mortality rate were lower in the Ig group compared with the control (9.9 vs. 12.8 days and 44.4% vs. 76.5%, respectively). Total time of hospitalization was decreased (13.3 vs. 15.9 days). The differences in this small study were not statistically significant. The observations were supported by a steady decrease in endotoxin levels after Ig treatment compared with control.
				VD		X		
				MOR		X		
				HOS		X		
				EA		X		
[149]	Severe infections with >90% IMV patients)	IgM/IgA-enriched Ig preparation (total dose 400 mL in 36 h)	104	VD	X			Overall, the number of VD was reduced significantly in the Ig group compared with the control (6.7 vs. 9.2 days) as well as in survivors (4.2 vs. 7.2 days) and in the high-risk postoperative “R3” group (5.5 vs. 12.7 days). Whereas the control group remained (mean) 21.5 days in ICU, those receiving Ig stayed only 14.8 days (*p* < 0.01).
				VDsg	X			
				HOSsg	X			
[150]	Septic shock within 24 h after onset of symptoms	IgM/IgA-enriched Ig preparation (250 mg/kg/day (20 mg/kg/h) for 3 consecutive days)	168	VFD		X		Marked improvement in lung function in the Ig group compared with control was observed in the overall population (median VFD: 22 vs. 12 days) and in the propensity sm population (*n* = 118 pairs; 20 vs. 10 days), although both were non-significant. Median number of ICU-free days increased markedly for Ig treatment (13 vs. 5 days) and in the sm population (13 vs. 4 days). Mortality rate was significantly reduced in the overall (25.0% vs. 46.1%, *p* = 0.004) and in the sm population and 25.4% vs. 45.8%, *p* = 0.021). See also Tables 2 and 3.
				VFDsm		X		
				MOR	X			
				MORsm	X			
[35]	Post-surgical intra-abdominal infections	IgM/IgA-enriched Ig preparation (total dose: 1300 mL over 3 days)	64	IMV		X		Although the difference was not significant, the duration of IMV was shorter in the Ig group. This effect was related to signs indicating a non-significant reduction in inflammation in the Ig group (reduction in the percentage of patients with fever, a larger decline in body temperature, a slightly larger decrease in TNF-α, endotoxin, and leukocyte levels, a stronger increase in platelets, and a faster and permanent decrease in PCT levels) and a shorter hospital stay (mean: 21 vs. 36 days).
				INF		X		
				EA		X		
				HOS		X		
[146]	Medical and surgical severe sepsis and septic shock (sepsis score: 12–27 and APACHE-II score 20–35)	IVIg (900 mg/kg total dose (600 mg/kg on day 0, 300 mg/kg on Day 1)) (low dose)	624	Lung			X	No beneficial effect of Ig treatment on pulmonary function was observed in the first 4 days, although the total duration of IMV overall and in the survivor subgroup was significantly shorter in the Ig group compared with the control group (mean: 11.3 vs. 13.4 days and 12.0 vs. 15.0 days, respectively). This study did not reveal a reduction in 28-day mortality by Ig treatment compared with the control (39.3% vs. 37.3%), but revealed a significantly higher ICU survival rate in the Ig group (60.7% vs. 54.5%). No effect of Ig treatment on plasma levels of IL-6 and TNF-receptors I and II was found. See also Table 3.
				VD	X			
				VDsg	X			
				MOR			X	
				SUR(ICU)	X			
				INF			X	

APACHE-II: acute physiology and chronic health evaluation II score; COVID-19: corona virus disease 2019; CRP: C-reactive protein; CT: computed tomography; d: number of days; EA: endotoxin activity; ICU: intensive care unit; INF: inflammatory markers; h: hours; HOS: hospitalization days; Ig: immunoglobulin IL: interleukin; IMV: invasive mechanical ventilation; IVIg: intravenous immunoglobulin G; MOR: mortality; N: total number of patients in the study; n: number of patients in the (sub)group analyzed;; PCT: procalcitonin; REF: reference; SARS-CoV: severe acute respiratory syndrome corona virus; sCAP: severe community-acquired pneumonia; sg: subgroup; sm: score-matched subgroup; SUR: survival; TNF: tumor necrosis factor; VD: ventilator days; VFD: ventilator-free days.

**In summary**, although neutrophils and their NETs provide a first line of defense against pathogens in the alveoli, excessive recruitment and activation can lead to bystander tissue damage and further loss of lung function [57,118,119,120]. Based on the different modes of action, the presence of sufficient amounts of polyvalent Igs may prevent the recruitment and tissue trafficking of large numbers of neutrophils into the alveoli and reduce NETs/NETosis by reducing chemokine secretion, the activity of anaphylatoxins, and the relevant signals for NETs/NETosis. Alveoli tissue protection is partially provided by IgG, and (novel) IgM-dependent mechanisms seem to complete this protection. Nonclinical studies have suggested a protective activity of Igs; similar effects were observed in patients with sCAP and sepsis, where improvements in alveolar functions were shown. Patients treated with Igs seem to recover more rapidly from lung dysfunction (measured as reduced time of hospitalization and/or invasive mechanical ventilation) than patients in control groups. Although these effects were not always statistically significant, they are of clinical relevance and point to the protective role of Igs in alveolar tissue damage that is caused by severe infections. This is supported by a recent meta-analysis, which included 15 randomized clinical trials (*n* = 712) and four cohort studies (*n* = 818) and concluded that the use of IgM/IgA-enriched Ig preparations shortened the length of invasive mechanical ventilation (mean difference −3.16 days; 95% CI −5.71 to −0.61) compared with the control groups [151]. Further large, multicenter studies are warranted to evaluate this respiratory benefit.

### 4.2. Vascular Events in Systemic Infection

The main function of the vascular endothelium is to provide adequate blood and oxygen supplies to tissues and organs. Additional functions include the production of chemoattractant compounds, the expression of adhesion molecules, the regulation of the translocation of neutrophils from blood into tissues, and the prevention of coagulation [152]. In cases of systemic infection, a strong activation of endothelial cells alters these functions, particularly through the interaction of endothelial cells and neutrophils (Figure 2B) [153].

#### 4.2.1. The Role of Neutrophils and NETs

In the case of sepsis, endothelial cell activation is induced by a synergistic action of inflammatory cytokines and bacterial toxins, causing the expression of adhesion molecules such as intercellular adhesion molecule-1 (ICAM-1), vascular cell adhesion molecule-I (VCAM-1), P-selectin, and E-selectin on the surface of endothelial cells [154]. The P- and E-selectin adhesion molecules bind to constitutively expressed integrin ligands on neutrophils, such as E-selectin-ligand-1 or P-selectin glycoprotein ligand (PSGL-1), which triggers the attachment of neutrophils to the endothelium [154]. Subsequent activation of neutrophils leads to expression of the integrin lymphocyte function-associated antigen-1 binding to the ICAM-1 and VCAM-1 adhesion molecules (Figure 2B) and crawling by expression of macrophage-1 integrin (Mac-1). 

In the presence of sepsis, the ability of neutrophils to deform in order to pass through capillaries becomes impaired. Due to proinflammatory mediators and bacterial products released into the vasculature during sepsis, leukocyte rigidity is increased [155], leading to neutrophils being trapped in the capillary beds of, e.g., the lungs or the kidney [156]. This may contribute to vascular occlusion and promote tissue ischemia [153].

Thus, strong induction of endothelial adhesion molecules, excessive neutrophil trafficking, and firm attachment of neutrophils to the endothelium are important steps in the early development of a vascular inflammatory reaction and endothelial dysfunction. The firm attachment of neutrophils to activated endothelial cells in sepsis and interactions with platelets promote the formation of NETs [157,158]. It was suggested that NETs provide a platform for the adhesion of platelets, red blood cells, and bacteria. However, NETs ultimately lead to endothelial damage in a histone- and myeloperoxidase-dependent manner and may also lead to thrombosis, thus compromising the perfusion of vital organs (Figure 2B). DNase treatment, which eliminated NETs, was shown to improve the survival rate of animals with sepsis [159,160].

#### 4.2.2. Nonclinical Studies Investigating the Supportive Role of Polyvalent Immunoglobulins on Vascular Protection 

Several in vitro studies investigated the anti-inflammatory effect of IVIg on endothelial cells. IVIg blocked TNF-α- and IL-β-induced expression of adhesion molecules (ICAM-1, VCAM-1) and chemokines (monocyte chemoattractant protein-1, granulocyte colony stimulating factor) by endothelial cells [161]. Furthermore, a high dose of IVIg inhibited the proinflammatory nuclear factor kappa-light-chain enhancer of the activated B-cells (NFκB) pathway in endothelial cells and reduced secretion of IL-6 and expression of adhesion molecules on the cell surface [162,163]. Although the effect on this NFκB signal transduction pathway was not reproducible in another study [164], a high dose of IVIg inhibited the secretion of cytokines such as IL-6 and granulocyte colony stimulating factor by coronary artery endothelial cells that were induced by TNF-α. 

Effects of IVIg on neutrophil adhesion on endothelial cells were evaluated in an in vivo microscopy experiment in the rat hepatic microvasculature in response to TNF-α, LPS, or after CLP [165,166]. When no IVIg was given, an increase in the number of neutrophils that adhered to the sinusoidal wall was observed. Perfusion of the sinusoids and Kupffer cell phagocytic activity were decreased. These effects were diminished by IVIg treatment, which upregulated the number of Kupffer cells in the sinusoids to prevent a hepatic microvascular inflammatory response during sepsis and endotoxemia in rats that was caused by neutrophil recruitment and accumulation [166].

In a multiple sclerosis mouse model, IVIg significantly reduced leukocyte adhesion and leukocyte rolling, causing a decrease in α4-integrin-dependent adhesion via VCAM-1 ligand. This was associated with an improvement of symptoms, as measured via a score in mice with experimental autoimmune encephalomyelitis [167]. In vitro, IVIg treatment reduced the adhesion of leukocytes, which were isolated from patients with multiple sclerosis, to human endothelial cells [167]. In contrast, IVIg treatment was not beneficial in an ischemia-reperfusion model—a model of brain inflammation [167]. In this study, neutrophils and platelets attached to intracranial vessels during reperfusion. Subsequent IVIg treatment enhanced this effect. Thrombosis and stroke are potential risks of IVIg treatment and might be related to the FcγRIIa expression on the platelets, expression of which was found to be increased after stroke [168,169].

In other in vivo studies, IVIg treatment seemed to inhibit FcγRIII and Src homolog 2 domain-containing protein tyrosine phosphatase 1 signaling, which prevented neutrophil adhesion and activation in areas of vascular injury [170]. IVIg was also found to reduce specific PSGL-1 antibody binding to leukocytes, thus altering the endothelial adhesion activity of these cells [171].

The microcirculatory actions of IVIg and IgM/IgA-enriched Ig preparations were compared in a hamster endotoxin model [172]. Following LPS injection, an induction of substantial leukocyte–endothelial cell interactions was observed. Increased microvascular leakage, decreased platelet counts, and capillary perfusion failure were observed in intravital microscopy. The study showed that IVIg and IgM/IgA-enriched Ig preparations reduced venular leakage and ameliorated the decrease in platelet count. In addition, the IgM/IgA-enriched Ig preparation significantly reduced leukocyte adhesion in venules, which was associated with normalization of capillary perfusion 24 h after induction of endotoxemia [172].

#### 4.2.3. Clinical Studies Investigating the Supporting Role of Polyvalent Immunoglobulins in Vascular Protection

The mechanism behind the protective effect of Igs on the vasculature in severe infections has not been studied directly in patients. To translate this activity from animal models to the clinic, surrogate endpoints such as the development of shock or the use of vasopressors can be used. Due to a reduction in IgM and/or IgG levels in sepsis, the opsonization of pathogens and the (opsono)phagocytosis activity are likely to be decreased. As a result, activation of the alternative and mannan-binding lectin complement pathways may lead to an increase in C5a, the generation of bacterial debris, and the overactivation of neutrophils. Vascular permeability or damage is caused by excessive translocation of neutrophils through the endothelium or by oxidative damage to the endothelium caused by the NETs. Consequently, the development of septic shock and the use of vasopressors in patients may indicate damage to the endothelial cells, i.e., an increase in vascular permeability.

A prospective, non-interventional study showed that septic patients with low IgG levels required more days of vasopressor therapy [8]. In line with these results, Dominioni and colleagues showed that fewer surgical patients with sepsis died from septic shock when patients were treated with IVIg compared with the untreated control group (2/29 patients vs. 11/33 patients) [173]. These results were confirmed in a larger study (*n* = 113) showing that the overall mortality of severely septic surgical patients (initial sepsis score ≥ 17) was significantly reduced following IVIg treatment compared with the control group (IVIg: 33% vs. control: 64%; *p* < 0.005) (Table 2, [174]).

Clinical investigations indicated that a decrease in IgM is a predominant characteristic in patients with severe sepsis who develop a septic shock, suggesting a possible protective role of IgM in this process. Reduced levels of IgM after the start of vasopressor therapy correlated with an unfavorable prognosis in morbidity [18]. In a prospective, randomized, controlled study in severe sepsis patients, IgM/IgA-enriched Ig preparation treatment caused a slight but clinically relevant reduction in the incidence of septic shock (IgM/IgA-enriched Ig preparation: 38% (8/21) vs. control: 57% (12/21)) (Table 2, [36]). This was related to a significantly faster decrease in PCT levels and a non-significant reduction in mortality rate compared with the control group. 

One retrospective case-control study in COVID-19 reported that treatment with an IgM/IgA-enriched Ig preparation significantly (*p* = 0.045) reduced the number of patients developing septic shock compared with the control group (12.5% (3/24) vs. 21.7% (7/23)) and, accordingly, the mortality rate (Table 2, [175]). 

In an exploratory study in 19 patients diagnosed with sepsis or septic shock, detailed analyses of the sublingual microcirculation using video microscopy and tissue oxygenation and of microvascular reactivity using spectroscopy showed that an IgM/IgA-enriched Ig preparation improved vascular perfusion in septic patients (Table 2, [176]). This resulted in better tissue oxygenation and preservation of organ functions, although the vasopressor dose was not different from that of the placebo group. 

The use of vasopressors was investigated in a retrospective study in a heterogeneous patient cohort with septic shock admitted to the ICU (Table 2, [150]). Although no significant difference in vasopressor-free days was observed (median: 24 days (IQR: 12–27 days)) compared with the control group (23 days (IQR: 3–27 days)), treatment with an IgM/IgA-enriched Ig preparation significantly reduced mortality rates overall compared with control treatment (25.0% vs. 46.1%; *p* = 0.004) and markedly increased the number of ICU-free days (13 days vs. 5 days). Similarly, in a prospective, randomized, controlled study (*n* = 160), where a comparable number of sCAP patients developed septic shock in the treatment groups (IgM/IgA-enriched Ig preparation: *n* = 35; placebo: *n* = 44). Overall, no difference in vasopressor-free days was detected (median: 14 days (IQR: 6–20 days) vs. 15 days (IQR: 8–20 days)) [23]. In another prospective study in patients with septic shock, only a trend for reduced vasopressor use was found after treatment with IgM/IgA-enriched Ig preparation (Table 2, [147]).

Igs are not known to be involved in the repair of vascular damage that is often found in patients with septic shock. The results of clinical studies reaffirm the importance of early treatment, i.e., before a patient develops septic shock. In addition, early treatment with Igs seems to be essential to prevent mortality after the onset of shock [150,177,178].

**Table 2:** Effect of Igs on vasculature parameter.

REF	Indication	Ig Preparation (Dose)	N	Effect	Significant Difference	Marked Change	No Difference	Study Results
[174]	Severe sepsis after surgery (sepsis score: 17–30)	IVIg (1000 mg/kg total dose (400 mg/kg on Days 0 and 1, and 200 mg/kg on Day 5)) (high dose)	113	Shock	X			Ig treatment resulted in a significant reduction in the number of patients developing and subsequently dying from septic shock (7% vs. 29%). Additionally, the ICU stay of survivors was markedly but not significantly reduced in patients receiving Ig compared with controls (mean: 19 vs. 26 days), and overall mortality was reduced significantly (33% vs. 64%). This study identified a subpopulation of septic patients that respond to high-dose Ig treatment.
				ICUd		X		
				MOR	X			
[36]	Severe sepsis	IgM/IgA-enriched preparation (250 mg/kg/day on 3 consecutive days)	42	Shock		X		Ig treatment resulted in a trend in the reduction of patients developing septic shock (38% vs. 57%). This vascular measure was supported by the fact that inflammation (PCT levels) was statistically significantly decreased in the Ig group compared with the control. Furthermore, the mortality was markedly but not significantly reduced in the Ig group in this small study (23.8% vs. 33.3%).
				INF	X			
				MOR		X		
[147]	Severe sepsis and septic shock	IgM/IgA-enriched preparation (5 mg/kg/day over 3 days)	26	VPT			X	No significant differences between or within the groups were detected for the use of vasopressor therapy. Nevertheless, a trend was observed, particularly on Day 3, where 45.4% of patients in the Ig group and 71.4% in the control group received vasopressors. See also Table 3.
				VPTt		X		
[175]	COVID-19	IgM/IgA-enriched preparation (500 mg/kg/day over 2 consecutive days)	47	Shock	X			Ig treatment resulted in a significant reduction in the number of patients developing septic shock (12.5% vs. 21.7%). Most patients developed acute kidney (80%) and liver failure (80%) at the time of ICU admission, before the infusion of Ig. Some renal complications occurred at a higher rate in the Ig group compared with controls. Markedly fewer patients in the Ig group developed pulmonary embolism (12.5% vs. 26.1%), sepsis (20.8% vs. 39.1%), and mortality was significantly lower (37.5% vs. 56.5%).
				PE		X		
				Sepsis		X		
				MOR	X			
[176]	Sepsis or septic shock within 24 h after onset of symptoms	IgM/IgA-enriched preparation (250 mg/kg/day over 3 consecutive days)	19	PVDt	X			Persistent microcirculatory alterations during septic shock are associated with organ failure and death. Ig was able to significantly increase (21.7 ± 4.7 to 25.5 ± 5.1 mm/mm^2^) the sublingual PVD after 72 h, whereas it was reduced in the placebo group (25 ± 5.8 to 20.7 ± 4.1 mm/mm^2^). The MFI was significantly increased at 24 h in the Ig group (median: 2.68 to 2.93 AU), while it significantly decreased at 72 h in the placebo group (median: 2.83 to 2.67 AU). Infusion of Ig may be associated with an increase in the sublingual microvascular density and blood flow quality. The administration of Ig did not induce any significant variation in MAP or HR and did not correlate with variations in hemodynamic parameters or cytokine levels.
				MFIt	X			
[150]	Septic shock within 24 h after onset of symptoms	IgM/IgA-enriched preparation (250 mg/kg/day (20 mg/kg/h) for 3 consecutive days)	168	VPFD			X	Nearly no improvement in the use of vasopressors was observed in the Ig group compared with the control group overall (median VPFD: 24 vs. 23 days) or in the propensity score-matched population (*n* = 118 pairs; 24 vs. 22 days), despite the reductions in the 30-day mortality rate in the Ig group, which were significant in the overall and score-matched populations (25.0% vs. 46.1% and 25.4% vs. 45.8%). See also Tables 1 and 3.
				VPFDsm			X	
				MOR	X			
				MORsm	X			

AU: arbitrary units; d: number of days; h: hours; COVID-19: coronavirus disease 2019; HR: heart rate; ICU: intensive care unit; Ig: immunoglobulin; INF: inflammatory markers; IVIg: intravenous immunoglobulin G; MAP: mean arterial pressure; MFI: microvascular flow index; MOR: mortality; N: number of patients in the study; n: number of patients in the (sub)group analyzed; PCT: procalcitonin; PE: pulmonary embolism; REF: reference; sm: score-matched subgroup; t: selected time point; VPFD: vasopressor-free days; VPT: vasopressor therapy; PVD: perfused vessel density.

**In summary**, vascular inflammatory reaction and endothelial dysfunction are not only caused by the pathogen and their toxins but also by impaired neutrophil functions. Nonclinical studies suggest that, in addition to toxin neutralization activity, anti-inflammatory activity, and modulation of complement activity, IVIg and IgM/IgA-enriched preparations may reduce vascular damage. Clinical data suggest that the timing of treatment with Igs is critical for efficacy and may better protect from vascular damage during sepsis if administered early, i.e., before septic shock has developed. When administered early, fewer patients developed septic shock and died. Delay of administration was found to increase the risk of death by 3% for every 24 h after the onset of shock [179]. These observations in patients with severe infections support a protective role of Igs in endothelial functions. 

## 5. Prevention of Coagulopathy and Organ Failure by Immunoglobulins

Dysregulated interactions between the endothelium, neutrophils, complement, and the coagulation system play a pivotal role in the pathogenesis of severe bacterial infections and severe viral infections such as COVID-19 [180,181]. Changes in major hemostasis pathways lead to an imbalance in procoagulation and anticoagulation pathways. This may result in the clinical symptoms seen in sepsis and septic shock, including excessive bleeding, thromboembolic events, or circulatory collapse due to (micro)thrombosis in the vasculature, causing organ dysfunction, MOF, and death. 

### 5.1. The Role of Endothelial Cells, Platelets, Neutrophils, and Complement

Endothelial cells contain anticoagulant agents such as thrombomodulin, heparan sulfate, and plasminogen activator. In healthy subjects, endothelial cells are nonadhesive and nonthrombogenic. During infection, these cells may be activated (e.g., by PAMPs or inflammatory cytokines or due to excessive neutrophil trafficking). Upon activation of endothelial cells, the endothelial cell surface changes from an antithrombotic to a prothrombotic surface, which results in upregulation of expression of tissue factor and von Willebrand factor [182]. Inhibitory signals (e.g., antithrombin, activated protein C, and fibrinolysis) are lost, which drives platelet adherence and activation [183].

The primary function of activated platelets is wound closure and limiting infection by trapping pathogens in local blood clots. Other functions of activated platelets include (1) induction of the acute-phase response to infection by secreting proteins such as cytokines and chemokines; (2) activation of the classical and alternative complement pathways; and (3) targeting neutrophil functions to drive host-immune responses. Together with neutrophils and complement, platelets can also fuel inflammation and tissue damage during systemic infection [184].

PAMPs such as LPS stimulate the interplay between neutrophils and platelets [185]. In neutrophils, this leads to activation of neutrophil integrins (e.g., Mac-1 that binds to platelet glycoprotein Ib*α*). In platelets, this leads to the expression of P-selectin that binds to neutrophil PSGL-1. When platelets bind to neutrophils, they also trigger the release of chemokines and the formation of NETs. Platelets thus collaborate with neutrophils to form NETs and both participate in pathogen capture via sequestration of pathogens within thrombi, which are entangled in the highly thrombotic NETs (reviewed in Davis et al. [186]). In turn, neutrophils secrete plasminogen activator inhibitor-1, which prevents the formation of plasmin and fibrinolysis as well as the degradation of the thrombus.

In parallel with these processes, the complement system comes into play. Via the endothelial cell-activated coagulation pathway components (particularly thrombin, coagulation factor [F]X, FXI, and plasmin), C3 and C5 are converted into C3a and C5a [187,188]. Activated platelets also induce the generation of anaphylatoxins on their surface [189]. Release of C3a/C5a and chemokines by both endothelial cells and platelets leads to the recruitment of neutrophils. Neutrophils attach via interaction with lymphocyte function-associated antigen-1 to the endothelium (via ICAM-1) and with PSGL-1 to the platelets (via P-selectin), leading to complex formation in the vasculature (Figure 2B). Stimulation of TLR and C5a receptor and interaction with platelets strongly stimulate neutrophil degranulation and release of reactive oxygen species and NETs. Also, C5a was found to induce expression of plasminogen activator inhibitor-1 by neutrophils [190]. All these interconnected processes lead to the stable formation of thrombi in the vasculature, which contain platelets, neutrophils, NETs, and complement factors.

In sepsis, these processes contribute to disseminated inflammatory signals and coagulopathy, with blood clot formation throughout the body. Blocking the microvasculature may lead to pulmonary embolisms, kidney dysfunction, cardiovascular complications, and even multiple organ dysfunctions (MODs) or MOF. Strong obstruction of blood vessels in patients with sepsis may lead to endotheliopathy-associated vascular microthrombotic disease and organ failure, which is an important and independent predictor of clinical outcome in patients with severe sepsis [191].

### 5.2. Nonclinical Studies Investigating the Supporting Role of Polyvalent Immunoglobulins 

Simultaneous control of inflammation, endothelial cell activation, complement activation, and coagulation could improve patient outcomes. As discussed in previous sections, Igs have multiple modes of action related to these processes. Obermayer et al. showed that, in particular, natural IgM was found to inhibit coagulation and thrombosis by binding to DAMPs on microvesicles [95]. These mechanisms may prevent endothelial cell activation, platelet activation, and excessive neutrophil recruitment and thus may also impinge on coagulation processes in the vasculature.

In vivo studies in rats showed that infusion with LPS causes disseminated intravascular coagulation (DIC) and hemostatic activation, with the pathophysiology of LPS-induced DIC mimicking the type of DIC observed in patients with sepsis [192]. Nonlethal LPS infusion in rats led to marked glomerular fibrin deposition, which was reduced by timely pretreatment with IVIg [193]. Plasma levels of thrombin–antithrombin complex (a measure of thrombin generation), TNF-α, and IL-6 were dose dependently decreased, and the drop in platelets and fibrinogen—the hallmark of consumption coagulopathy—was attenuated. Moreover, liver and kidney organ dysfunctions (indicated by alanine aminotransferase and creatinine levels), as well as glomerular fibrin deposition, were all ameliorated by IVIg. Suppression of hemostatic activation and microthrombi formation by IVIg might thus attenuate organ damage caused by DIC [193].

Igs can affect platelet activity indirectly. A study showed an influence of Igs on the exotoxin of *Streptococcus pneumonia* pneumolysin, which causes lysis of erythrocytes and platelets [194,195]. IVIg counteracted pneumolysin-induced lysis of platelets. Note that IgM/IgA-enriched preparations also contain antibodies that neutralize pneumolysin. Although not statistically significant, platelet counts increased in *Streptococcus pneumonia*-infected sCAP patients treated with an IgM/IgA-enriched preparation (*n* = 15) compared with the placebo group (*n* = 18) [195].

### 5.3. Clinical Studies Investigating the Supporting Role of Polyvalent Immunoglobulins

#### 5.3.1. Prevention of Coagulopathy 

The international normalized ratio, activated partial thromboplastin time, platelet count, and fibrinogen concentration are clinical markers that have been used to investigate changes in hemostasis after treatment with Igs. 

The effect of IVIg on coagulation markers was investigated in a small, retrospective study in sepsis patients (Table 3, [30]). IVIg was given within two days after hospitalization. It should be noted that treatment with IVIg was performed in a statistically significantly higher proportion of patients with a more severe condition, as indicated by a higher Sequential Organ Failure Assessment (SOFA) score (9.1 ± 3.7 vs. 6.5 ± 3.7, *p* < 0.05) and lower IgG baseline levels (i.e., IgG: 854 ± 380 mg/dL) compared with the control group (IgG: 1333 ± 736 mg/dL) (*p* < 0.01). In IVIg-treated patients, sepsis-induced hemostasis imbalance was improved as shown by significant decreases from baseline (*p* < 0.01) in the international normalized ratio, activated partial thromboplastin time, plasminogen activator inhibitor-1, CRP, IL-6, and PCT levels. In the control group, statistically significant decreases from baseline were only seen for the thrombin–anti-thrombin complex and IL-6 levels (*p* < 0.05 and *p* < 0.01, respectively). IVIg treatment stabilized hypercoagulability, effectively inhibited thrombin production, improved fibrinolysis suppression, and significantly improved the DIC (*p* < 0.05) and SOFA scores (*p* < 0.01) relative to baseline. Although not statistically significant, 28-day mortality was markedly reduced (5.3%, 1/19 patients) compared with the control group (18.2%, 4/22 patients).

Effects on coagulation parameters were also investigated in severe sepsis patients treated with IgM/IgA-enriched Ig preparations (Table 3, [147]). Four days after treatment, platelet and fibrinogen concentrations were significantly higher (*p* = 0.016 and *p* = 0.017, respectively) in the IgM/IgA-enriched Ig preparation group than the control group. Platelet counts fluctuated around baseline during the first treatments with the IgM/IgA-enriched Ig preparation, which was followed by an increase above baseline on Day 4. In the control group, a steady decline in platelet counts was observed over the four days, and counts dropped below the lower level of normal. Platelet count decline may be caused by lysis due to exotoxins or clot formation. After four days, platelet counts and fibrinogen concentrations were significantly higher in the IgM/IgA-enriched Ig preparation group, suggesting less clot formation. A reduced time needed for organ support was observed in the IgM/IgA-enriched Ig preparation group compared with the control group (60% vs. 79% of subjects on mechanical ventilation, 40% vs. 64% on dialysis on Day 4), but these results were not statistically significant. No significant difference in viscoelastic or aggregometric measurements was observed between groups. In this study, patients were enrolled at a late stage of disease, with a mean Acute Physiology and Chronic Health Evaluation II (APACHE-II) score of 35, and presumably suffered from advanced systemic inflammation for a longer period. As discussed earlier, in the advanced stages of sepsis and septic shock, dysregulation of hemostasis may already be profound, and the effects on viscoelastic variables cannot be reversed by Igs. This is supported by a study performed in the rat DIC model [193].

Apart from the positive effects of Igs in preventing coagulation, it should be noted that some IVIg drug insert packages include a warning about the risk of thromboembolic events such as myocardial infarction, cerebral vascular accident (including stroke), pulmonary embolism, and deep vein thromboses. The inclusion of a warning is mandated by the United States Food and Drug Administration. At-risk patients include, e.g., those with paralysis, venous stasis due to obesity or permanent immobility, or a history of thromboembolic events. In addition, other comorbidities, including hypertension, diabetes, and advanced age, are associated with the risk of thromboembolism [196,197,198]. This increased risk for thromboembolic events is possibly related to a relative increase in blood viscosity through the high influx of Igs. This could perhaps promote erythrocyte or platelet aggregation. Other laboratory assessments revealed that coagulation factor XIa seems to be co-purified with IgG in some IVIg manufacturing processes. The risks and benefits of IVIg administration should be considered before treatment initiation, as should the possibility of lowering the dose and infusion rates to reduce the risk of thromboembolic events. 

#### 5.3.2. Prevention or Improvement of Organ Dysfunction 

Coagulopathy can result in vascular microthrombotic disease, causing MODs or MOF. The APACHE-II score is a measure of the degree of organ damage, dysfunction, or failure. Therefore, in addition to mortality rates, this score can be used as a clinical endpoint to investigate the effect of Ig treatment. 

Although clinical studies report heterogeneous results for IVIg therapy, several studies have shown improvement in MODs and/or APACHE-II scores after IVIg treatment [199,200,201,202]. Other studies showed that dose as well as disease stage at the time of administration (high APACHE-II score) seem to play an important role [38,146,203]. In a small, prospective, randomized study in patients with early septic shock and severe respiratory failure, treatment with an IgM/IgA-enriched Ig preparation did not improve MODs within eight days compared with standard therapy (Table 3, [38]). The importance of dose and administration time point was confirmed in a systematic review and meta-analysis [134].

In contrast, in another prospective study in septic shock patients who received an IgM/IgA-enriched Ig preparation, a statistically significant decrease in the APACHE-II score five days after the start of treatment (*p* < 0.05) and a significant decrease in the 6-week mortality rate (3.7% vs. 32.1%; *p* = 0.082) was observed compared with the control group (Table 3, [204]). This was associated with a significant decrease in endotoxin levels within 24 h of treatment (*p* < 0.01). Higher endotoxin levels were shown to be associated with a higher APACHE-II score [205].

Yavuz and colleagues retrospectively investigated the effect of an IgM/IgA-enriched Ig preparation in patients with sepsis-induced MODs by evaluating the change in APACHE-II score (Table 3, [206]). Despite a longer ICU stay in the IgM/IgA-enriched Ig preparation group, the general condition of patients based on APACHE-II scores improved (statistically non-significant) to a greater extent in this group (27 to 16) compared with the control group (27 to 23) 4 days after the start of treatment. Sepsis and septic shock remain the most important triggers of acute renal failure in ICU patients, with a much higher mortality rate in patients with sepsis than in patients without sepsis. Although the number of patients with renal dysfunction was higher in the IgM/IgA-enriched Ig preparation group in the Yavuz study [206], creatinine levels improved significantly from baseline on Day 4 (*p* = 0.013), indicating a positive effect on renal function compared with the control group, where no improvement was observed. Since the 28-day case mortality rate was significantly decreased with an IgM/IgA-enriched Ig preparation (24/56 patients) compared with the control group (53/62 patients) (*p* < 0.0001), patients in the control group may have died from MODs [206].

Similarly, in another retrospective study, in patients with septic shock, a higher percentage of patients required renal replacement therapy in the IgM/IgA-enriched Ig preparation group (30.4% vs. 22.4%; *p* = 0.29, Table 3, [150]). However, in this study, mortality was significantly reduced (*p* = 0.004), and fewer patients died from MODs (17.4% (16/92 patients) vs. 28.9% (22/76 patients)) or refractory shock (5.4% (5/92 patients) vs. 13.2% (10/76 patients)). It should be noted that acute kidney injury was found to be related to the presence of sucrose in some IVIg preparations and is more frequent in patients with risk factors for acute kidney injury.

The efficacy of an IgM/IgA-enriched Ig preparation in severe sepsis patients was found to depend on the APACHE-II score at the start at treatment [207]. In a prospective study, APACHE-II scores were 21.27 ± 7.23 in the IgM/IgA-enriched Ig preparation group and 23.5 ± 7.91 in the control group. Mortality rate was significantly reduced from 40% in the control group to 22.4% in the IgM/IgA-enriched Ig preparation group. In patients with an APACHE-II score of 20–29, mortality rate was 22.2% after treatment with the IgM/IgA-enriched Ig preparation and 55% in the control group. Taking into account the heterogeneity of the disease, selecting patients by APACHE-II score may help to identify patient subgroups that may benefit from Ig treatment [207]. In addition, in a peritonitis study, patients with intermediate APACHE-II scores (11–15) benefited the most from the IgM/IgA-enriched Ig preparation treatment, as was shown by a markedly reduced length of hospital stay (IgM/IgA-enriched Ig preparation: 22 days ± 14 days vs. control: 55 days ± 41 days) [35].

**Table 3:** Effect of Igs on coagulation parameters and organ dysfunction measures.

REF	Indication	Ig Preparation (Dose)	N	Effect	Significant Difference	Marked Change	No Difference	Study Results
[30]	Sepsis	IVIg (1500 mg total dose (5.0 g/day for 3 days)) (dose regimen in Japan)	41	COAG	X			After IVIg treatment, a significant decrease compared with baseline was observed for various coagulation/fibrinolysis molecular markers (PT-INR (mean: 1.6 to 1.2), APTT (44.5 to 29.3), SF (30.1 to 14.7), PAI-1 (182 to 16.5)), markers for inflammation (CRP (mean: 16.3 to 9.9), PCT (32.2 to 6.3)), as well as the JAAM DIC score. Decrease of these markers after treatment was not significant for the control group. In both groups, only IL-6 (22943 to 85.5 vs. 1214 to 55) and TAT (8.2 to 3.0 vs. 20.5 to 3.7) decreased significantly after treatment. The DIC (*p* < 0.05) and SOFA (*p* < 0.01) scores significantly decreased after IVIg treatment compared with baseline. The 28-day mortality rate was markedly lower in the Ig group compared with the control (5.3% vs. 18.2%).
				INF	X			
				DIC	X			
				SOFA	X			
				MOR		X		
[147]	Severe sepsis and septic shock	IgM/IgA-enriched preparation (5 mg/kg/day over 3 days)	26	EAt	X			Endotoxin levels influence coagulation parameters. On Day 1, endotoxin was significantly decreased 6 and 12 h after Ig treatment and differed significantly compared with the control group following 6 h of treatment (0.26 ± 0.07 vs. 0.43 ± 0.07). The platelet count in the Ig group was significantly higher on Day 4 (mean: 200/nL vs. 87/nL). The fibrinogen concentration was significantly higher on Day 2 (475 mg/dL vs. 311 mg/dL, *p* = 0.016) and on Day 4 (420 mg/dL vs. 307 mg/dL, *p* = 0.017) compared with the control group. The levels of inflammatory markers and viscoelastic or aggregometric measures did not differ between groups. Regarding organ dysfunctions/failure, a marked change in the decrease in the SOFA score was observed with Ig treatment on Day 4 (mean: 11.7 to 7.0 vs. 10.6 to 9.5), and the use of dialysis increased in the control group (35.7% to 64.3% on Day 4) as compared with the Ig group (33.3% to 40%).
				COAG	X			
				INF			X	
				SOFA		X		
				MOD		X		
[204]	Gram-negative (endotoxemia) septic shock, <24 h after onset of symptoms	IgM/IgA-enriched preparation (Day 1: 600 mL; Days 2 and3: 300 mL)	55	EAt	X			Endotoxemia in patients with septic shock has physiological and biological consequences, resulting in higher APACHE-II scores. Ig treatment was associated with a significant decrease in endotoxin levels within 24 h of treatment, which was associated with a statistically significant decrease (*p* < 0.05) in the APACHE-II score after Day 5. In the control group, APACHE-II scores further increased until Day 10. This was related to a significant decrease in the 6-week mortality rate compared with the control group (1/27 (3.7%) vs. 9/28 (32.1%), *p* = 0.0082).
				ASt	X			
				MOR	X			
[146]	Medical and surgical severe sepsis and septic shock (sepsis score: 12–27 and APACHE-II score 20–35)	IVIg (900 mg/kg total dose (600 mg/kg on Day 0, 300 mg/kg on Day 1)) (low dose)	624	Lung			X	Regarding organ dysfunction, 90.5% of patients were mechanically ventilated at baseline. No beneficial effect of Ig treatment on pulmonary function was observed (see Table 1). A significantly stronger decline of the disease severity sepsis score (−1.21) and a more pronounced decline in the APACHE-II score (-1.25) in the Ig group compared with placebo were found. The improvement in APACHE-II was mainly due to improved GCS and decrease in leukocytes. The 28-day mortality rate was not significantly different between groups (39.3% in the Ig group vs. 37.3% in the control group), although the ICU survival rate was significantly higher in the Ig group (60.7% vs. 54.5%).
				VD	X			
				VDsg	X			
				SS	X		X	
				AS	X			
				MOR			X	
				SUR(ICU)	X			
[202]	STSS	IVIg (2000 mg/kg total dose (1 g/kg on Day 1 and 0.5 g/kg on Days 2 and 3)) (high dose)	21	SOFAt	X			Ig treatment resulted in improvement of organ dysfunction during treatment Days 1–3 in the Ig group, as assessed by a significant decrease in SOFA score. The resolution of shock in survivors (88 h vs. 122 h, not significant) was faster in the Ig group. The 28-mortality was 3.6-times higher in the placebo group (10% vs. 36%, not significant) and higher after 180 days (20% vs. 36%). Ig treatment significantly increased the neutralizing activity against superantigens.
				Shock		X		
				MOR		X		
				SA	X			
[38]	Septic shock accompanied by severe respiratory failure	IgM/IgA-enriched preparation (5 mL/kg/day via 8 h infusion over 3 days)	33	CRP	X			No significant resolution of organ failures was observed. Only a trend for length of IMV (median VD: 13 vs. 17). Markers of organ dysfunction and inflammation (platelets, PT, total protein, albumin, WBC) were nearly identical, except for CRP levels, which were significantly lower in the Ig group on Days 4, 5, and 6. Median length of ICU stay was not significantly different, but a decrease was observed in the Ig group (median: 15 vs. 26 days). The 28-day mortality rates were nearly identical in the two groups (25.0% vs. 29.4%). In this small study, at late-stage sepsis, organ dysfunction was likely driven by the host response rather than the infective pathogen itself.
				INFt			X	
				MOD			X	
				VD		X		
				MOR			X	
				ICUd		X		
[206]	Sepsis-induced MODs	IgM/IgA-enriched preparation (5 mg/kg/day over 3 days)	118	AS		X		Many factors affect mortality from MODs, including the number of affected organs and the degree of organ dysfunction. In this study, all patients had 2 or 3 organ dysfunctions, mostly respiratory and cardiovascular. Although APACHE-II scores decreased significantly on Day 4 in both the Ig (*n* = 56; from 27 to 16) and the control groups (*n* = 62; from 27 to 23), the effects were more pronounced in the Ig group. Ig treatment significantly increased the length of ICU stay (mean: 36 vs. 22 days), but decreased the overall (85.5% vs. 42.9%) and 28-day case fatality rate (69.3% vs. 25.0%).
				MOR	X			
[150]	Septic shock within 24 h after onset of symptoms	IgM/IgA-enriched preparation (250 mg/kg/day (20 mg/kg/h) for 3 consecutive days)	168	VFD		X		Improvement in organ dysfunction with Ig, although non-significant, was observed for the lung compared with control in the overall population (median VFD: 22 vs. 12 days) and in the propensity sm population (*n* = 118 pairs; 20 vs. 10 days), although both were non-significant. This was despite the fact that more patients required renal replacement therapy in the Ig group compared with the control group (overall: 30.4% vs. 22.4% and in sm: 28.8% vs. 20.3%). Sepsis and septic shock are the most important triggers of acute renal failure with a high mortality rate. However, the 30-day mortality was reduced by 21.1% (overall: 25.0% vs. 46.1%) in the Ig group despite the higher number of patients requiring renal replacement. See also Tables 1 and 2.
				VFDsm		X		
				MOR	X			
				MORsm	X			

APACHE-II: Acute Physiology and Chronic Health Evaluation II score; AS: APACHE-II score; APTT: activated partial thromboplastin time; COAG: coagulation; CRP: C-reactive protein; d: number of days; DIC: disseminated intravascular coagulation score; EA: endotoxin activity; h: hour; GCS, Glasgow Coma Scale; ICU: intensive care unit; Ig: immunoglobulin IL-6: interleukin 6; IMV: invasive mechanical ventilation; INF: inflammatory markers; JAAM: Japanese Association for Acute Medicine; MODs: multiple organ dysfunction; MOR: mortality; N: total number of patients in the study; n: number of patients in the (sub)group analyzed; PAI-1: plasminogen activator inhibitor-1; PCT: procalcitonin; PT, prothrombin time; PT-INR: platelet count, prothrombin time– international normalized ratio; REF: reference; SA: neutralizing activity against superantigens; SF: soluble fibrin; sg: subgroup; sm, score-matched subgroup; SOFA: Sequential Organ Failure Assessment score; SS: sepsis score, STSS: streptococcal toxic shock syndrome; SUR: survival; t: selected time point; TAT: thrombin–antithrombin complex; VD: ventilator days; VFD: ventilator-free days; WBC: white blood cell.

**In summary**, during infection, the surface of activated endothelial cells changes from an antithrombotic to a prothrombotic surface. Interactions between neutrophils, platelets and complement contribute to coagulopathy. Blocking the microvasculature may ultimately lead to MODs or MOF. Nonclinical data suggest that the benefits observed in severely infected patients treated with Igs may be related to their ability to prevent or affect coagulation processes by various mechanisms, as summarized above. If administered early, i.e., before extensive damage has occurred, Ig therapy seems to improve and/or prevent organ dysfunctions/failure that may occur during sepsis progression. This may well lead to a faster recovery and reduce mortality rates. Results from clinical studies are supported by encouraging data from various meta-analyses, suggesting a reduction in mortality in adults with sepsis and septic shock treated with IgM/IgA-enriched preparations [133,134,151,208,209,210,211,212,213].

## 6. Conclusions and Future Use of Immunoglobulins

The published nonclinical and clinical data discussed in this review indicate that timely administration of Igs could prevent secondary tissue damage to the alveoli, which could otherwise contribute to lung edema and increase the risk for invasive mechanical ventilation and systemic spread of the lung infection. Several small clinical studies have shown a clear trend toward a decreased number of ventilator days, faster recovery, and/or shorter hospitalization times when Ig preparations were used. Results were not statistically significant in most studies, however, most studies were not powered to show statistically significant differences. In line with this, a recent meta-analysis showed that the use of IgM/IgA-enriched preparations significantly shortened the length of mechanical ventilation [151].

Early administration of polyvalent Igs may also prevent secondary vascular tissue damage. Inappropriate activation and homing of neutrophils to the microvasculature contribute to the pathological manifestations of sepsis. Protection from excessive complement activation, anaphylatoxin scavenging, and dampening the cytokine storm may all be effective mechanisms to prevent vascular events (e.g., progression to septic shock) and coagulation processes (e.g., progression to coagulopathy), in which neutrophils, the complement system, and endothelium seem to play major roles. Additional nonclinical and clinical studies are warranted to further investigate the interactions between Igs and neutrophils, complement, endothelium, and coagulation factors. 

Due to the lack of clear effects on mortality in studies conducted in heterogeneous target populations, the use of Ig treatments is currently not recommended by sepsis and sCAP guidelines. This may change when more promising results become available from studies performed in, e.g., less heterogeneous patient populations. So far, a few studies have been conducted with polyvalent Ig preparations in well-defined and more homogenous patient populations with various types of severe infections. These studies provided supportive, statistically significant results—including reduction in mortality—in patients with postoperative abdominal sepsis [214], Gram-negative septic shock [204], meningitis [215], streptococcal toxic shock syndrome [216], sepsis on invasive mechanical ventilation [217], severe infection plus a hyper-inflammatory response [23,218,219], early severe sepsis or early septic shock [147,150], patients with defined APACHE-II scores [207], or low IgM levels [23,220,221], or the use of TO-Predisposition Insult Response and Organ Failure score to select a homogenous patient cohort and start treatment in a timely manner [209]. Additional studies with more restricted inclusion and exclusion criteria, which would result in a better-defined study population, are needed and may provide a better understanding of the efficacy of Igs.

In addition to the target population, the dose and type of Ig treatment seem to be important, as shown in studies with IgM/IgA-enriched preparations in Gram-negative sepsis [213], or in studies using high-dose IVIg administration in toxic shock syndrome [202,216,222]. A meta-analysis concluded that IVIg treatment, if dosed at 1.5–2 g/kg, is likely to reduce the all-cause mortality of patients with sepsis [132]. Similarly, for IgM/IgA-enriched Ig preparations, a recent meta-analysis indicated that mortality rates in pediatric and neonatal patients with sepsis were significantly lower in children who received treatment with the IgM/IgA-enriched Ig preparation compared with controls (OR = 0.41; 95% CI 0.32–0.55). In neonatal studies, a higher treatment duration (>3 days) resulted in a higher total dose administered as compared with the recommended dose (OR = 0.32; 95% CI 0.22–0.47 vs. OR = 0.61; 95% CI 0.41–0.92) [223]. Investigations of the effect of Ig dosing on different baseline Ig concentrations may help to further define the appropriate dose to be used in severe infectious diseases. Selecting a personalized dose that aims to achieve a specific IgM serum concentration threshold is a novel approach that is currently under investigation in septic shock patients [221]. A personalized dose may decrease mortality rates more effectively than a standard dose, which may not reach the threshold required for efficacy in all patients.

Thus, for future trials, the dose and type of Ig administered, time point of administration, target population, and efficacy endpoints should be well considered. Results will help to better select the right type of Ig preparation and define the correct dose and administration time for the patient. 

## Data Availability

Not applicable.
